# Preoperative risk factors associated with anastomotic leakage after colectomy for colorectal cancer: a systematic review and meta-analysis

**DOI:** 10.1590/0100-6991e-20223363-en

**Published:** 2022-11-10

**Authors:** VINÍCIUS EVANGELISTA DIAS, PEDRO ALVES SOARES VAZ DE CASTRO, HOMERO TERRA PADILHA, LARA VICENTE PILLAR, LAURA BOTELHO RAMOS GODINHO, AUGUSTO CLAUDIO DE ALMEIDA TINOCO, RODRIGO DA COSTA AMIL, ALEIDA NAZARETH SOARES, GERALDO MAGELA GOMES DA CRUZ, JULIANA MARIA TRINDADE BEZERRA, THAIS ALMEIDA MARQUES DA SILVA

**Affiliations:** 1 - Faculdade Santa Casa BH, Programa de Pós-graduação Stricto Sensu em Medicina - Biomedicina - Belo Horizonte - MG - Brasil; 2 - Universidade Iguaçu - Itaperuna - RJ - Brasil; 3 - Faculdade Metropolitana São Carlos - Bom Jesus do Itabapoana - RJ - Brasil; 4 - Faculdade de Medicina da Universidade Federal de Minas Gerais - Belo Horizonte - MG - Brasil; 5 - Hospital São José do Avaí, Departamento de Cirurgia Geral - Itaperuna - RJ - Brasil; 6 - Universidade Estadual do Maranhão, Centro de Estudos Superiores de Lago da Pedra - Lago da Pedra - MA - Brasil; 7 - Universidade Estadual do Maranhão, Programa de Pós-Graduação em Ciência Animal - São Luís - MA - Brasil

**Keywords:** Risk Factors, Anastomotic Leaks, Colon Surgery, Colon Diseases, Fatores de Risco, Fístula Anastomótica, Neoplasias Colorretais, Cirurgia Colorretal

## Abstract

**Introduction:**

anastomotic leak (AL) after colectomy for colorectal cancer (CRC) is a life-threatening complication. This systematic review and meta-analysis aimed to evaluate the preoperative risk factors for AL in patients submitted to colectomy.

**Methods:**

the bibliographic search covered 15 years and 9 months, from 1st January 2005 to 19th October 2020 and was performed using PubMed, Cochrane Library, Scopus, Biblioteca Virtual em Saúde, Europe PMC and Web of Science databases. The inclusion criteria were cross-sectional, cohort and case-control studies on preoperative risk factors for AL (outcome). The Newcastle-Ottawa scale was used for bias assessment within studies. Meta-analysis involved the calculation of treatment effects for each individual study including odds ratio (OR), relative risk (RR) and 95% confidence intervals (95% CI) with construction of a random-effects model to evaluate the impact of each variable on the outcome. Statistical significance was set at p<0.05.

**Results:**

cross-sectional studies were represented by 39 articles, cohort studies by 21 articles and case-control by 4 articles. Meta-analysis identified 14 main risk factors for AL in CRC patients after colectomy, namely male sex (RR=1.56; 95% CI=1.40-1.75), smoking (RR=1.48; 95% CI=1.30-1.69), alcohol consumption (RR=1.35; 95% CI=1.21-1.52), diabetes mellitus (RR=1.97; 95% CI=1.44-2.70), lung diseases (RR=2.14; 95% CI=1.21-3.78), chronic obstructive pulmonary disease (RR=1.10; 95% IC=1.04-1.16), coronary artery disease (RR=1.61; 95% CI=1.07-2.41), chronic kidney disease (RR=1.34; 95% CI=1.22-1.47), high ASA grades (RR=1.70; 95% CI=1.37-2.09), previous abdominal surgery (RR=1.30; 95% CI=1.04-1.64), CRC-related emergency surgery (RR=1.61; 95% CI=1.26-2.07), neoadjuvant chemotherapy (RR=2.16; 95% CI=1.17-4.02), radiotherapy (RR=2.36; 95% CI=1.33-4.19) and chemoradiotherapy (RR=1.58; 95% CI=1.06-2.35).

**Conclusions:**

important preoperative risk factors for colorectal AL in CRC patients have been identified based on best evidence-based research, and such knowledge should influence decisions regarding treatment.

## RESUMO

**Objetivo:**

fístula anastomótica (FA) após colectomia para câncer colorretal (CCR) é complicação grave. Esta revisão sistemática e meta-análise avaliou os fatores de risco pré-operatórios para FA em pacientes submetidos à colectomia.

**Métodos:**

a pesquisa bibliográfica abrangeu 15 anos e 9 meses (1 de janeiro de 2005 - 19 de outubro de 2020), sendo utilizadas as plataformas PubMed, Cochrane Library, Scopus, Biblioteca Virtual em Saúde, Europe PMC e Web of Science. O critério de inclusão foram estudos transversais, coorte e caso-controle em fatores de risco pré-operatórios para FA (desfecho). A escala Newcastle-Ottawa foi usada para avaliação de viés dos estudos. A metanálise envolveu o cálculo dos efeitos de tratamento para cada estudo individualmente incluindo *odds ratio* (OR), risco relativo (RR) e intervalo de confiança de 95% (IC95%) com construção de modelo de efeitos aleatórios, para avaliar o impacto de cada variável (p<0,05).

**Resultados:**

foram selecionados 39 estudos transversais, 21 coortes e quatro casos-controle. A metanálise identificou 14 fatores de risco para FA em pacientes com CCR após colectomia, que são sexo masculino (RR=1,56; IC 95%=1,40-1,75), tabagismo (RR=1,48; IC 95%=1,30-1,69), alcoolismo (RR=1,35; IC 95%=1,21-1,52), diabetes mellitus (RR=1,97; IC 95%=1,44-2,70), doenças pulmonares (RR=2,14; IC 95%=1,21-3,78), doença pulmonar obstrutiva crônica (RR=1,10; IC 95%=1,04-1,16), doença coronariana (RR=1,61; IC 95%=1,07-2,41), doença renal crônica (RR=1,34; IC 95%=1,22-1,47), altas notas na escala ASA (RR=1,70; IC 95%=1,37-2,09), cirurgia abdominal prévia (RR=1,30; IC 95%=1,04-1,64), cirurgia de emergência (RR=1,61; IC 95%=1,26-2,07), quimioterapia neoadjuvante (RR=2,16; IC 95%=1,17-4,02), radioterapia (RR=2,36; IC 95%=1,33-4,19) e quimiorradioterapia (RR=1,58; IC 95%=1,06-2,35).

**Conclusões:**

importantes fatores de risco pré-operatórios para FA colorretais em pacientes com CCR foram identificados com base nas melhores pesquisas baseadas em evidências e esse conhecimento deve influenciar decisões relacionadas ao tratamento.

## INTRODUCTION

The surgical procedure of choice for the treatment of resectable non-metastatic colorectal cancer (CRC) is colectomy with lymph node removal. Anastomotic leakage (AL) constitutes a serious complication of low anterior resection for rectal cancer, leading to increased risk of postoperative morbidity, protracted hospitalization and the likely need for additional surgical procedures that may affect the quality of life of the patient^1^. The incidence of AL is typically within the range 7.5 to 10.4%^2^, and evolution of the condition is believed to be associated with a number of specific risk factors.

According to previous studies, the predictors of AL are male sex, coronary artery disease, type of surgical procedure, elevated serum albumin, low rectal anastomosis and neoadjuvant radiotherapy^3^ ^,^ ^4^. Knowledge of the key factors that predispose patients to develop AL is important in determining the most suitable time for surgery, in the early diagnosis of complications and in the management of pre- and post-operative care.

Considering the severity of AL and the divergent views in the literature concerning the most significant predictors of this life-threatening complication, we carried out a systematic review and meta-analysis with the aim of determining the preoperative risk factors associated with the evolution of the condition in patients submitted to colectomy for CRC.

## METHODS

### Protocol and registration

This systematic literature review formed part of a study submitted to and approved by the Research Ethics Committee of Santa Casa de Belo Horizonte under the protocol CAAE 36476320.2.0000.5138. The Ethics Committee waived the requirement of written informed consent since the systematic review and meta-analysis were based entirely on data published in the literature. The review was carried out in accordance with the checklist included in the Preferred Reporting Items for Systematic Reviews and Meta-Analyses (PRISMA 2020) protocol and was registered on the International Prospective Register of Systematic Reviews (PROSPERO) database (https://www.crd.york.ac.uk/prospero) under protocol CRD42020219325.

### Information sources and search strategy

**Figure 1:**
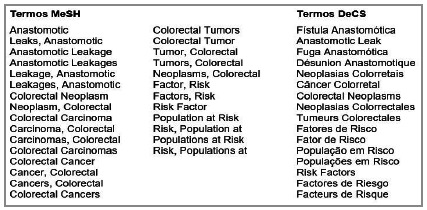
Keywords used in the bibliographic search.

The bibliographic search, which covered the 15 year and nine months period from 1st January 2005 to 19th October 2020, was performed on 17th November 2020 and updated on 1st December 2021 without new entries. Studies were retrieved from the PubMed, Cochrane Library, Scopus, Biblioteca Virtual em Saúde, Europe PMC and Web of Science databases using combinations of DeCS and MeSH descriptors (Figure 1 ; Table 3).

### Eligibility criteria and selection of studies

Cross-sectional, cohort, case-control, and randomized controlled studies relating to the risk factors of AL in patients submitted to colectomy (right hemicolectomy, extended right hemicolectomy, high left segmental colectomy, left colectomy, sigmoid colectomy, subtotal colectomy, total colectomy, high anterior resection, low anterior resections, ultra-low anterior resection) for CRC were considered eligible for inclusion.

The exclusion criteria were studies published in languages other than English, Portuguese, Spanish or French, literature reviews, systematic reviews, meta-analyses, studies without original data, case reports, case series, animal studies, and grey literature.

**Figure 2:**
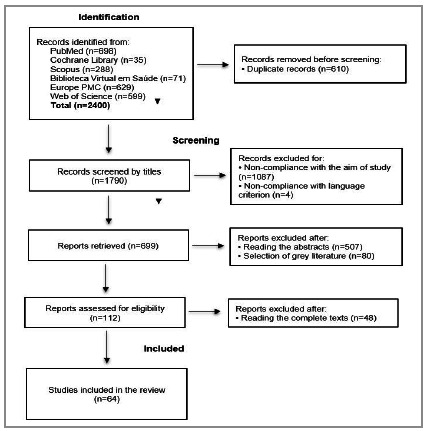
Fluxograma dos estudos incluídos.

Relevant publications were selected by two researchers (VED and LVP) on the basis of the eligibility criteria by reading the titles and, subsequently, the detailed abstracts. Duplicate studies were eliminated and the full texts of the remaining articles were analyzed to select the studies to be included in this review. In cases of disagreement about the inclusion of a publication, consensus was attained by discussion or through mediation with the help of a third researcher (LBRG). The PRISMA 2020 flow chart shown in Figure 2 outlines the phases of the selection of studies.

### Data extraction and data quality

Data were extracted from the selected studies independently by two researchers (HTP and PASVC) according to the Population, Exposure, Comparison, Outcome and Study design (PECOS) approach and the information compared. For each selected study, details regarding the authors, study design, date, number and characteristics (region/country of origin, sex, age and underlying medical conditions) of the participants, statistical methods employed in the analysis of data, calculation of sample size and study outcome were recorded using an Excel spreadsheet. The Newcastle-Ottawa Scale (NOS) was used to assess the risk of bias and the quality of the studies employed in the meta-analyses. This tool comprises 8 items categorized within 3 domains, namely selection of study groups, comparability of the groups and outcome. The maximum aggregate score across the domains was 9, according to which a score of 7 - 9 indicated high quality, 4 - 6 suggested a moderate risk of bias, and 0 - 3 a high risk of bias. In the present study, an average score of 6 or above was considered satisfactory^5^.

### Statistical analysis

All analyses were performed using RevMan software version 5.4 (Cochrane, London, UK). Treatment effects, including odds ratio (OR), relative risks (RR) and 95% confidence intervals (95%CI), were computed for each selected study and, in cases where mean values and standard deviations for a given risk factor were provided, mean risk differences between patients with and without AL were calculated. Cochran’s Q test and I^2^ statistics were employed to quantify the variability (heterogeneity) among the results of the selected studies with the significance level set at p<0.10. The degree of heterogeneity was interpreted according to the range of I^2^ as follows: 0 - 40%, likely not important; 30 - 60%, moderate; 50 - 90%, significant; and 75 - 100% substantial. In consideration of the considerable heterogeneity identified among the studies, meta-analyses were conducted using the random-effects model to evaluate the impact of each variable on the outcome. The statistical significance of the differences between groups in the univariate analysis was set at p<0.05.

## RESULTS

### Overall features of the studies

The bibliographic search resulted in 64 studies being selected for inclusion in the review (Figure 2; Table 1). The total sample population comprised 184,110 participants of which 17,342 (9.42%) exhibited AL. Cross-sectional studies were represented by 39 articles^1^ ^,^ ^2^ ^,^ ^6^ ^-^ ^42^, cohort studies by 21 articles^3^ ^,^ ^43^ ^-^ ^62^ and case-control by four articles^63^ ^-^ ^66^, and the groups of studies of each design type presented satisfactory mean NOS scores, i.e. 5.05, 6.62 and 5.25, respectively^5^. Considering studies of all design types together, 26.6% (17/64) could be classified as high quality according to the NOS scale, while 64% (41/64) presented a moderate risk of bias and 9.4% (6/64) exhibited a high risk of bias.

**Table 1:** Articles included in the systematic review and their respective Newcastle-Ottawa Scale (NOS) quality assessment scores.

Articles	Location	Total sample population	NOS score	Reference
Cross sectional studies				
Kinugasa et al., 2020	Kurume, Japan	97	6	[1]
Zhou et al., 2019	Beijing, China	288	5	[2]
Bakker et al., 2014	Leiden, Netherlands	15,667	6	[6]
Chen et al., 2011	Shantou, China	750	5	[7]
Choi et al., 2006	Hong Kong, China	1417	3	[8]
Frasson et al., 2015	Valencia, Spain	3139	6	[9]
Articles	Location	Total sample population	NOS score	Reference
Fukada et al., 2019	Gifu, Japan	101	3	[10]
Gong et al., 2014	Nanjing, China	460	5	[11]
Hayden et al., 2015	Maywood, EUA	123	7	[12]
Iancu et al., 2008	Cluj-Napoca, Romania	993	5	[13]
Jung et al., 2008	Seoul, South Korea	1391	5	[14]
Kang et al., 2013	Irvine, EUA	72055	5	[15]
Kato et al., 2019	Tokyo, Japan	447	4	[16]
Kawada et al., 2014	Tokyo, Japan	154	5	[17]
Kim et al., 2009	Seul, South Korea	270	5	[18]
Krarup et al., 2012	Copenhagen, Denmark	9333	8	[19]
Kruschewski et al., 2007	Berlin, Germany	276	3	[20]
Kryzauskas et al., 2020	Vilnius, Lithuania	900	9	[21]
Kumar et al., 2011	Nothern, India	108	5	[22]
Kwak et al., 2017	Seul, South Korea	423	5	[23]
Lai et al., 2013	Guangzhou, China	1312	5	[24]
Lee et al., 2020	Seul, South Korea	4282	7	[25]
Lee et al., 2018	Hwasun, South Korea	1063	4	[26]
Lee et al., 2008	Seul, South Korea	1278	4	[27]
Liu et al., 2016	Chengdu, China	306	6	[28]
Maeda et al., 2015	Osaka Japan	201	5	[29]
Majbar et al., 2014	Rabat, Morocco	130	4	[30]
Martel et al., 2008	Ottawa, Canada	220	5	[31]
Nickelsen et al., 2005	Nordre Ringvej, Denmark	5181	6	[32]
Peeters et al., 2005	Leiden, Netherlands	924	6	[33]
Piecuch et al., 2015	Zabrze, Poland	222	3	[34]
Rudinskaite et al., 2005	Kaunas, Lithuania	269	3	[35]
Shen et al., 2019	Beijing, China	423	6	[36]
Suzuki et al., 2021	Ube, Japan	136	7	[37]
Tian et al., 2017	Beijing, China	11397	5	[38]
Vermeer et al., 2014	Netherlands	517	6	[39]
Wang & Liu, 2020	Beijing, China	496	5	[40]
Xu & Kong, 2020	Shenyang, China	382	5	[41]
Yang et al., 2013	Nanjing, China	753	5	[42]
Cohort studies				
Zhou et al., 2018	Guangzhou, China	956	8	[3]
Akasu et al., 2010	Tokyo, Japan	120	8	[43]
Akiyoshi et al., 2011	Tokyo, Japan	1,146	7	[44]
Bisgård et al., 2013	Herlev, Denmark	2755	8	[45]
Ciorogar et al., 2017	Cluj-Napoca, Romania	378	6	[46]
Articles	Location	Total sample population	NOS score	Reference
Eriksen et al., 2005	Oslo, Norway	1958	8	[47]
Gustafsson et al., 2015	Visby, Sweden	3428	8	[48]
Hu & Cheng, 2015	Chongqing, China	1968	5	[49]
Ionescu et al., 2013	Cluj-Napoca, Romania	252	8	[50]
Jannasch et al., 2015	Magdeburg, Germany	17867	8	[51]
Liu et al., 2018	Guangzhou, China	646	7	[52]
Matsuda et al., 2016	Tokyo, Japan	179	4	[53]
Nisar et al., 2012	Cleveland, EUA	1862	6	[54]
Nordholm-Carstensen et al., 2019	Roskild, Denmark	1414	7	[55]
Park et al., 2016	Seul, South Korea	10477	7	[56]
Reilly et al., 2014	Dublin, Ireland	129	3	[57]
2015 European Society of Coloproctology Collaborating Group, 2020	Valência, Spain	2444	6	[58]
Tanaka et al., 2017	Osaka, Japan	395	6	[59]
Voron et al., 2019	Paris, France	1025	7	[60]
Warschkow et al., 2011	St. Gallen, Switzerland	527	6	[61]
Yamamoto et al., 2012	Tokyo, Japan	111	6	[62]
Case-control studies				
Altin & Alkan, 2019	Istambul, Turkey	302	4	[63]
Asteria et al., 2008	Florence, Italy	520	6	[64]
Jestin et al., 2008	Uppsala, Sweden	372	5	[65]
Nishigori et al., 2014	Chiba, Japan	176	6	[66]

The included studies analyzed 16 different risk factors of AL, namely age, sex, smoking and drinking habits, tumor location, diabetes mellitus, lung disease, chronic obstructive pulmonary disease (COPD), coronary artery disease (CAD), chronic kidney disease (CKD), American Society of Anesthesiologists (ASA) physical status grade, previous abdominal surgery, CRC-related surgical emergency, neoadjuvant chemotherapy, radiotherapy and chemoradiotherapy. Results of the analyses of the impact of these risk factors on the critical outcome are presented below with the summary results of the meta-analyses summarized in Table 2. Among the 14 risk factors, only male sex in cohort studies reached heterogeneity higher than 75%, but in cross sectional and case control researches, heterogeneity was lower. Nevertheless, this finding must be analyzed with caution.

**Table 2:** Meta-analyses and summary statistics.

Risk factor	Type of study	Number of studies	Number of participants	RR (95%CI)	References	Heterogeneity
Sex	Cohort	15	36,284	1.42 (1.07 - 1.89)	[43,44,46-49, 54-62]	I² = 83%
Sex	Case-control	4	1,346	1.28 (1.02 -1.60)	[63-66]	I² = 0%
Sex	Cross-sectional	34	145,509	1.56 (1.40 - 1.75)	[2,6-24,26, 27,29-31, 33-35,37-42]	I² = 66%
Smoking habits	Cohort	5	21,180	1.48 (1.30 - 1.69)	[43,51,55,57,58]	I² = 0%
Alcohol consumption	Cross-sectional	9	77,567	1.35 (1.21 - 1.52)	[8,13,15,20,23, 24,31,40,41]	I² = 0%
Diabetes mellitus	Cross-sectional	16	11,871	1.97 (1.44 - 2.70)	[2,8-11,13, 16,20,21, 23,24,29, 34,40-42]	I² = 69%
Lung diseases	Cross-sectional	5	5,260	2.14 (1.21 - 3.78)	[9,13,16, 23,31]	I² = 42%
COPD	Cross-sectional	6	74,459	1.10 (1.04 - 1.16)	[2,8,15, 20,29,34]	I² = 0%
CAD	Cross-sectional	6	3,065	1.61 (1.07 - 2.41)	[2,7,8,20, 34,41]	I² = 27%
CKD	Cross-sectional	4	74,819	1.34 (1.22 - 1.47)	[8,15, 16,21]	I² = 0%
ASA grades	Cross-sectional	16	35,727	1.70 (1.37 - 2.09)	[2,6,8,9,18,19, 21,23,24,26, 27,31,35,39-4]	I² = 60%
Previous abdominal surgery	Cohort	4	13,417	1.30 (1.04 - 1.64)	[56,58,59,62]	I² = 0%
CRC-related emergency surgery	Cross-sectional	5	29,546	1.61 (1.26 - 2.07)	[6,8,9,13,19]	I² = 58%
Neoadjuvant chemotherapy	Cohort	5	15,610	2.16 (1.17 - 4.02)	[44,55-58]	I² = 42%
Neoadjuvant radiotherapy	Cohort	4	14,426	2.36 (1.33 - 4.19)	[47,54,56,57]	I² = 68%
Neoadjuvant chemoradiotherapy	Cross-sectional	10	6,902	1.58 (1.06 - 2.35)	[2,12,14,24, 26,27,31, 37,41,42]	I² = 41%

### Age

Forty-six studies had analyzed age as a risk factor for AL, but only six^11^ ^,^ ^14^ ^,^ ^22^ ^,^ ^42^ ^,^ ^56^ ^,^ ^64^ found a statistically significant difference (p<0.05) in advanced age (elderly patients) as a possible risk factor. In the meta-analysis, 3,727 patients were evaluated. Participants aged 60 years or less were compared to those aged over; an RR of 0.79 (95% CI: 0.58-1.08) was found, and only one^22^ of the studies showed a statistically significant difference. Thus, it is understood that age is not a risk factor for AL.

### Sex

Among the 53 studies that analyzed sex as a possible predictor of outcome, 22 revealed significant differences between males and females regarding the risk of AL. The parameter sex was examined in 15 cohort studies, of which six^47^ ^,^ ^54^ ^,^ ^56^ ^-^ ^58^ ^,^ ^59^ demonstrated increased risk of AL in males. Among four control cases^63^ ^-^ ^66^, only one^65^ showed a statistically significant difference. Out of the 34 cross-sectional studies, only 13 established higher risk of AL in male patients. Since one article^21^ separated data on colonic (sigmoid) from rectal surgeries, its inclusion in the systematic review had been done considering this division.

### Smoking habits

Fourteen studies included in the literature survey evaluated smoking as a risk factor for AL and, of these, nine^3^ ^,^ ^11^ ^,^ ^13^ ^,^ ^20^ ^,^ ^26^ ^,^ ^31^ ^,^ ^42^ ^,^ ^51^ ^,^ ^55^ showed significant differences between smokers and non-smokers. Smoking was assessed in nine cross-sectional studies involving 6,268 patients, but only two studies^13^ ^,^ ^31^ reported a statistical difference. A further five cohort studies addressed tobaccoism and two^51^ ^,^ ^55^ showed significant differences between the groups.

### Alcohol consumption

Thirteen studies included in the systematic review investigated alcoholism as a risk factor for AL although a significant difference between alcohol users and non-users was observed in only one cohort study^51^. The consumption of alcohol was examined by nine cross-sectional studies in the meta-analysis, and in this assessment a statistical difference was observed in only one study^15^.

### Tumor location

Regarding the tumor site, 15 articles evaluated this variable in relation to the occurrence of AL. Of this sample, nine studies^6^ ^,^ ^9^ ^,^ ^10^ ^,^ ^14^ ^,^ ^16^ ^,^ ^18^ ^,^ ^44^ ^,^ ^46^ ^,^ ^60^ showed a statistically significant difference (p<0.05). Of these, four studies^10^ ^,^ ^18^ ^,^ ^44^ ^,^ ^46^ suggested cancer located in the middle and lower rectum to be an independent risk factor for the occurrence of AL.

This meta-analysis analyzed the tumor site by comparing tumors on the right side (cecum, right colon, hepatic flexure and transverse colon) to the left side (splenic flexure, left colon and sigmoid). As the tumor location is a variable reported in the literature as an important risk factor for AL, a meta-analysis was performed comparing the left and right sides even though only three transversals studies^6^ ^,^ ^8^ ^,^ ^9^ have contemplated this variable in a feasible way. The meta-analysis of these three studies, which analyzed a total of 20,277 patients, did not show a statistically significant difference; the RR found was 0.89 (95% CI: 0.74, -1.07).

Tumors in the upper rectum were considered as those located 5cm above the anal margin; Tumors below 5cm were considered to be in the lower rectum. Four cross-sectional studies were accepted for the meta-analysis, two of which had a statistically significant difference^10^ ^,^ ^14^. However, the result of the meta-analysis showed RR 1.73 (95% CI: 0.95-2.03), not conceiving the tumor site in the lower or upper rectum as a risk factor for AL.

### Diabetes mellitus

**Figure 3:**
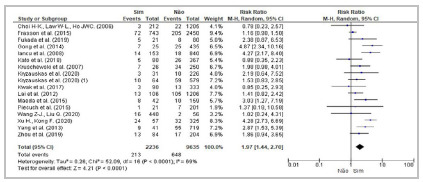
Forest Plot - Meta-analysis: Risk factor diabetes (cross-sectional studies). 95%CI: 95% Confidence Interval.

Twenty-five studies included in the review investigated diabetes as a risk factor for AL, of which six^11^ ^,^ ^13^ ^,^ ^29^ ^,^ ^41^ ^,^ ^42^ ^,^ ^49^ established a significantly higher risk of the outcome in diabetic patients compared with their non-diabetic counterparts. Meta-analysis of 16 studies confirmed diabetes as a risk factor (Table 2; Figure 3).

### Lung disease

Seven studies investigated lung disease as a risk factor for AL, three of which^9^ ^,^ ^13^ ^,^ ^43^ demonstrated significant differences between patients with and without the condition. Meta-analysis of five cross-sectional studies revealed a significant association between lung disease and increased risk of the outcome (Table 2).

### COPD

Six studies reported statistical differences between patients with and without COPD but in only one case-control study^63^ was the difference significant. In the meta-analysis of six cross-sectional studies that examined COPD as a risk factor for AL, one study^15^ presented a weighting of 98.9% by virtue of the large number of participants (72,055) involved (Table 2).

### CAD

Eight studies compared individuals with and without CAD, and four^2^ ^,^ ^7^ ^,^ ^20^ ^,^ ^63^ reported significant differences between the two groups regarding the evolution of AL. Of the six cross-sectional studies (Table 2) included in the meta-analysis, only one^2^ was able to demonstrate the association between CAD and increased risk of AL.

### CKD

The possibility of CKD as a risk factor for AL was investigated in six studies but no significant differences between patients with and without the disease were found in the univariate analysis of these reports. However, meta-analysis of four cross-sectional studies^8^ ^,^ ^15^ ^,^ ^16^ ^,^ ^21^ revealed that CKD was a predictor of the outcome (Table 2).

### ASA grade

**Figure 4:**
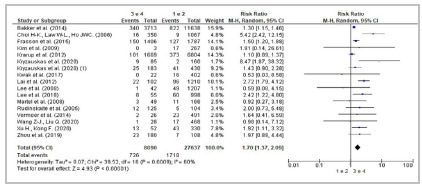
Forest Plot - Meta-analysis: Risk factor ASA (cross-sectional studies). 95%CI: 95% Confidence Interval.

Among the 33 studies that investigated ASA grades as risk factors for AL, 12^6^ ^,^ ^8^ ^,^ ^9^ ^,^ ^12^ ^,^ ^19^ ^,^ ^21^ ^,^ ^24^ ^,^ ^26^ ^,^ ^41^ ^,^ ^51^ ^,^ ^54^ ^,^ ^65^ demonstrated significant differences among individuals with dissimilar ASA classifications. Of the 16 cross-sectional studies selected for meta-analysis (Table 2; Figure 4), seven^6^ ^,^ ^8^ ^,^ ^9^ ^,^ ^21^ ^,^ ^24^ ^,^ ^26^ ^,^ ^41^ reported significant associations between high ASA grades and increased risk of AL.

### Previous abdominal surgery

Among the 13 studies comparing the development of AL in individuals who had or had not been submitted to abdominal surgery prior to colectomy for CRC, none showed statistical differences between the groups. Of the four cohort studies submitted to meta-analysis (Table 2), only one^56^ demonstrated a significant association between previous abdominal surgery and higher risk of AL.

### CRC-related surgical emergency

**Figure 5:**
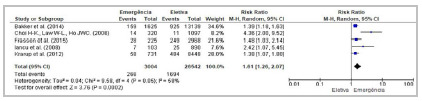
Forest Plot - Meta-analysis: Risk factor emergency surgery (cross-sectional studies). 95%CI: 95% Confidence Interval.

Twelve studies investigated CRC-related surgical emergency as a risk factor for AL and, of these, five studies^6^ ^,^ ^8^ ^,^ ^9^ ^,^ ^57^ ^,^ ^63^ demonstrated statistical differences between individuals that had required this procedure and those that had not. Meta-analysis of five cross-sectional studies^6^ ^,^ ^8^ ^,^ ^9^ ^,^ ^13^ ^,^ ^19^ established a significant association between CRC-related surgical emergency and higher risk of the outcome (Table 2; Figure 5).

### Neoadjuvant chemotherapy

**Figure 6:**
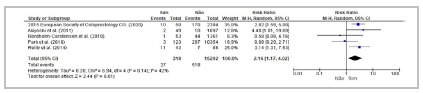
Forest Plot - Meta-analysis: Risk factor neoadjuvant chemotherapy (cohort studies). 95%CI: 95% Confidence Interval.

Among the 13 studies that investigated neoadjuvant chemotherapy as predictor of AL, three^23^ ^,^ ^34^ ^,^ ^63^ demonstrated statistical differences between individuals that had been submitted to the treatment and those that had not. Meta-analysis of five studies^44^ ^,^ ^55^ ^-^ ^58^ confirmed neoadjuvant chemotherapy as a risk factor. (Table 2; Figure 6).

### Neoadjuvant radiotherapy

**Figure 7:**
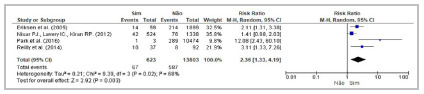
Forest Plot - Meta-analysis: Risk factor neoadjuvant radiotherapy (cohort studies). 95%CI: 95% Confidence Interval.

Three^30^ ^,^ ^47^ ^,^ ^57^ of the 10 studies that investigated neoadjuvant radiotherapy as a risk factor for AL demonstrated statistical differences between individuals that had been submitted to the treatment and those that had not. Of the four cohort studies considered in the meta-analysis (Table 2; Figure 7), three^47^ ^,^ ^56^ ^,^ ^57^ reported significant associations between radiotherapy and higher risk of AL.

### Neoadjuvant chemoradiotherapy

Thirteen studies investigated chemoradiotherapy as a risk factor for AL, and four of these^2^ ^,^ ^28^ ^,^ ^56^ ^,^ ^57^ demonstrated statistical differences between individuals that had been submitted to the treatment and those that had not. Of the ten cross-sectional studies considered in the meta-analysis (Table 2), two^2^ ^,^ ^27^ reported significant association between chemoradiotherapy and higher risk of AL.

## DISCUSSION

The physiopathological reasons for increased risk of AL in patients submitted to surgical treatment of CRC have yet to be elucidated, although it is recognized that this life-threatening condition is multifactorial^4^. The literature review presented herein disclosed 16 potential risk factors for AL, and 14 of these were confirmed in the subsequent meta-analysis.

Advanced age is no longer considered a contraindication for CRC surgery^67^ and our meta-analysis verified that older adults presented no increased risk of developing AL. Thus, it is accepted that primary anastomosis may be performed on senior patients without exposing them to increased risk of AL provided that they do not exhibit other comorbidities. It is likely that the improvement in operative techniques over the years, the spread of laparoscopy and the greater pre- and intraoperative care of elderly patients justify this result. Besides, preoperative selection of elderly patients may have resulted in the selection of the most apposite ones. The surgeons are probably more careful while choosing elderly patients for surgery treatment. These facts could explain why age isn’t considered as a risk factor for AL.

Several studies have demonstrated significant differences between males and females regarding the risk of AL and our meta-analysis confirmed that males exhibit a higher risk than females. Since men have a narrower pelvis, dissection of the tissues is more difficult and may cause postoperative complications. Furthermore, hormonal differences may influence intestinal microcirculation and, consequently, healing of the anastomosis^68^.

Our meta-analysis confirmed that smoking is a significant predictor of AL in CRC patients. Adequate tissue perfusion is essential for healing, and this seems to be particularly relevant for surgeries involving low rectal anastomosis^69^. The association between smoking and AL may be explained by four possible mechanisms, namely nicotine-induced vasoconstriction, cellular hypoxia caused by carbon monoxide, tissue hypoxia resulting in decreased collagen deposition and increased platelet adhesion and aggregation^70^.

A multicentre study^51^ has demonstrated that alcohol abuse is an independent risk factor for the evolution of AL (OR = 1.63; 95%CI = 1.23-2.15; p=0.001). Individuals who consume more than 35 drinks per week have a significantly higher risk of developing AL compared with those who abstain from alcoholic consumption. The probable causes are subclinical heart failure, immunosuppression and low hemostatic function^70^, all of which impair wound healing. However, the negative effect of alcohol on wound healing has yet to be proven^51^.

The meta-analysis did not verified tumor height as a risk factor for AL. Presumably, this occurred because many studies had shown divergences regarding the tumor height classification, which prevents the aggregation of these data in a meta-analysis. In addition, few studies have found this variable as an independent risk factor for the occurrence of AL, probably due to the lack of specific studies in this area. More studies are needed to elucidate this subject.

A meta-analysis performed by Rojas-Machado et al.^71^ showed that diabetes mellitus is a risk factor (OR = 1.60; 95%CI = 1.12 - 2.13) for AL, as confirmed by the results of our study. However, the association between the disorder and AL remains controversial since a large prospective study was unable to demonstrate that the presence of diabetes increased the rate of AL^72^. Nevertheless, the mortality rate among diabetic patients who developed AL was more than four-fold higher in comparison with their non-diabetic counterparts.

The impact of CAD on the development of anastomotic leak is unclear. An early study performed by Fawcett et al.^73^ demonstrated that microvascular disease at the serous layer of the anastomotic site increases the risk of leakages because defective microcirculation reduces blood flow and leads to poor wound healing. Considering that CAD is caused by atherosclerosis, our meta-analysis suggests that individuals with this condition may have simultaneous microvascular disease that interrupts circulation at the site of anastomosis. However, such assertions require further elucidation through properly conducted histopathological investigations.

Regarding ASA grades, our meta-analysis corroborated previous studies reporting similar levels of risk associated with physical status as, for example, OR = 1.71; 95% CI = 1.09 - 2.674 and OR = 1.76; 95% CI = 1.39 - 2.2371. Hence, patients classified as ASA grades III-IV, i.e. those who have a severe or life-threatening systemic disease in addition to cancer, are at serious risk of AL following CRC-related surgery.

This meta-analysis confirmed that CRC-related emergency surgery is a significant predictor of AL and showed risk levels that were similar to those reported by Rojas-Machado et al.^71^, namely OR = 1.96; 95%CI = 1.49 - 2.59. Increased risk of leakage in such cases may be explained by several underlying problems such as large blood loss, comorbidities, poor clinical condition of the individual and increased technical difficulty, all of which are superimposed on the same subject. Unfortunately, the risk of AL is cumulative and encompasses all of the risk factors applicable to the individual. Patients with comorbidities who require emergency resection generally suffer considerable blood loss, require transfusion and administration of vasoactive drugs and, in such cases, anastomosis is actually contraindicated^68^.

A number of retrospective studies^27^ ^,^ ^47^ ^,^ ^61^ ^,^ ^74^ ^,^ ^75^ have established that neoadjuvant radiotherapy with or without concurrent chemotherapy is a strong predictor of AL, a finding that is confirmed by the meta-analyses presented herein. However, according to Park et al.^75^, while chemoradiotherapy was a risk factor for AL in a subgroup of patients who did not receive a protective stoma after low rectal anterior resections for CRC, when all the patients submitted to the surgery were analyzed together, chemoradiotherapy did not appear as a risk factor. In a systematic review, McDermott et al.^68^ reported increased rates of AL and mortality in patients who underwent colon anastomosis for chronic radiation enteritis, suggesting that the operating surgeon should consider a previous history of irradiation in order to assess whether anastomosis is safe. In view of the data presented in that review and the results of our meta-analysis, surgeons should seriously consider performing a protective stoma during anastomosis in CRC patients undergoing neoadjuvant chemoradiotherapy, radiotherapy and chemotherapy.

Our study highlights the importance of further research in two main areas: (i) elucidation of the histo- and patho-physiological basis of AL in order to properly define and easily recognize this potentially fatal condition; (ii) understanding the underlying mechanisms by which the various risk factors influence the evolution of AL so that an optimal number of factors could be selected and ranked to facilitate decision-making about the management of risk development of the condition.

One limitation of our study was that some risk factors and their associations with the development of AL have been poorly investigated in patients colectomized for treatment of CRC. Additionally, some potential predictors studied by other authors had to be excluded from the meta-analysis since the variables could not be aggregated owing to heterogeneity between, and lack of standardization of, the same risk factors.

## CONCLUSION

Our meta-analysis identified 14 main risk factors for AL in patients colectomized for the treatment of CRC, namely male sex, smoking, alcohol consumption, diabetes mellitus, lung diseases, COPD, CAD, CKD, high ASA grades, previous abdominal surgery, CRC-related emergency surgery, neoadjuvant chemotherapy, neoadjuvant radiotherapy, neoadjuvant chemoradiotherapy. The risk factors with the highest RR of developing AL were: neoadjuvant radiotherapy, neoadjuvant chemotherapy, lung diseases, diabetes mellitus, and high scores on the ASA scale. Age and tumor location were not recognized as significant predictors of AL.
